# Comparative analyses of PAP smear data in pre and postmenopause Indian women

**DOI:** 10.6026/97320630016452

**Published:** 2020-06-30

**Authors:** Anbumozhi Kannan, Wangol Kiyam, Giridharan Bupesh, Matcha Bhaskar, Priavadhana Rajan Prasaad, Bheema Rao

**Affiliations:** 1 Department of Pathology, Sree Balaji Medical College and Hospital (SBMCH), BIHER, Chrompet, Chennai - 600044, India; 2 Research and Development Wing, Central Research Laboratory, Sree Balaji Medical College and Hospital (SBMCH), BIHER, Chrompet, Chennai - 600044, India; 3 Department of Zoology, Sri Venkadeswara University, Tirupathi, India

**Keywords:** Carcinoma cervix, PAP smear, premenopausal, postmenopausal

## Abstract

PAP smear is one of the best screening tools available for early detection of cervical cancer. Hence, we conducted a retrospective study at the Department of Pathology, Sree Balaji Medical College and Hospital over a period of one year by collecting PAP smear data. A total of 978 smears were collected out of which 59% were premenopausal and 41% were post menopausal women. Data shows that the prevalence of pre malignant and malignant lesions were more among the women of post menopausal age group than the pre menopausal age group.

## Background

Carcinoma cervix worldwide accounts for 15% of all cancers diagnosed in women [1]. Globally, cervical cancer is one of the most common cancers in women, with an estimate of 440,000 new cases annually. Eighty percent of these cases occurred in developing and under developed countries [2]. According to National Cancer Registry Program of India, it is evaluated that in India, roughly 100,000 ladies create cervical cancer growth every year. Cervical cancer is a preventable disease in the vast majority of women. Fortunately, the natural history of cervical cancer is such that it is possible to detect it early during a pre invasive curable stage by screening and early intervention thereby preventing progression into life threatening illness [3]. PAP smear is one of the best screening tools available for preventing cervical cancer. The most widely used system for describing PAP smear is The Bethesda System, 2001 [4]. PAP smear cervical cytology with sensitivity of 72% and specificity of 94% is suitable for population based screening programme [5]. Death rate from cervical cancer steadily increases with age. The mortality rate of cervical cancer will be reduced if screening is done between the ages of 40-45 years [6].

## Materials and Methods:

This was a retrospective study of all cervical PAP smear cases reported at the Department of Pathology, Sree Balaji Medical College and Hospital, Chennai, India for a period of 1 year from October 2015 to September 2016. Smears were taken via specialist doctors using changed Ayres wooden spatula, which was inserted and rotated 360o over cervix. Both ectocervix and endocervix were sampled. Samples were received in coplin jar (2 smears) dipped in isopropyl alcohol from the Dept of Obstetrics and Gynaecology, Sree Balaji Medical College and Hospital. Two smears were prepared, one stained with Haematoxylin and Eosin and other with PAP stains. All the smears were evaluated and subsequently examined and reported as per guidelines of the Bethesda system.

### Ethical Clearance on the use of data from human subjects:

This study was conducted under the supervision of the research ethical committee at Sree Balaji Medical College and Hospital, Tamilnadu, India.

## Results

A total of 978 cases were reported in the above-mentioned period and all the cases were analysed. The age of the patients ranged from 24 yrs to 79 yrs., of which 578 women (59%) were in pre menopausal age group and 400 women (41%) were in postmenopausal age group.

## Premenopausal age group:

**Figure 1:**
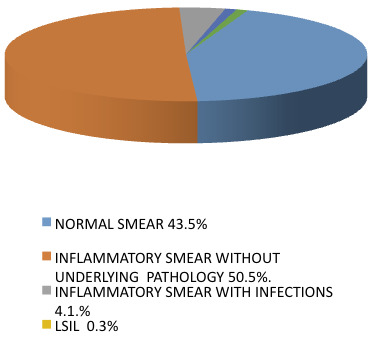
PAP smear data in premenopausal women

Among 978 women, 578 cases of women fell under the pre menopausal age Group. This group showed 43.5% (252) normal PAP smears. The inflammatory smear 50.5% (292) does not have any underlying pathology was seen in the investigated cases. Sexually Transmitted Diseases (STDs) caused by Trichomonas vaginalis, Candida albicans and Bacterial vaginosis were also diagnosed in the cytology smears.Bacterial vaginosis was seen in 6 women (1.0%), Trichomoniasis was found in 3 women (0.5%), candidiasis was found in 15 women (2.6%). LSIL was diagnosed in 6 (1.0%) women, HSIL was seen in 2 women (0.3%), and squamous cell carcinoma was found in 2 (0.3%) women (Figure 1).

## Postmenopausal Age Group:

**Figure 2:**
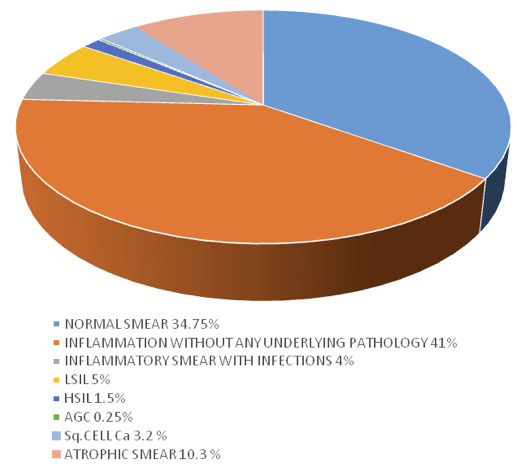
PAP smear data in postmenopausal women

Among 978 women, 400 of them fell in the postmenopausal category. Normal PAP smear results were found in 139 (34.75%)Postmenopausal women. Similarly, 164 cases (41%) of Inflammatory smear without any underlying pathology was found in STDs. Candidiasis were seen in 9 (2.2%) cases and bacterial vaginosis was seen in 7 (1.8%) cases. LSIL was diagnosed in 20 (5%) women and HSIL was seen in 6 women (1.5%). Atypical Glandular Cells was found in 1 case (0.25%) and squamous cell carcinoma was found in 13 (3.2%) woman. Atrophic smear was seen in 41 (10.3%) cases (Figure 2).

## Discussion:

Cancer of the cervix has been the most important cancer among women in the past two decades. In India the peak age for cervical cancer incidence is 55–59 years. Current data from the National Cancer Registry Program (NCRP) indicates that the most common sites of cancer among women are the breasts and the cervix [7].Cancer cervix is considered to be an ideal gynaecological malignancy for screening as it meets both test and disease criteria for screening. It has a long latent phase during which it can be detected as identifiable and treatable premalignant lesions which precede the invasive disease and the benefit of conducting screening for carcinoma cervix exceeds the cost involved [8]. Conventional PAP smears were used throughout this study. During conventional PAP smearing, drying artifacts, inadequate fixations, background materials and thick smears are frequently present.

The prevalence of LSIL is 4.8 times higher in the postmenopausal age group than the premenopausal age group in this data. Similarly the prevalence of HSIL and Squamous cell carcinoma are also 1.5 times and 3 times more in the postmenopausal age group women respectively. This is in concordance with Jones et al. [9] Elhakeem et al.. [10] also recorded a progressive increase in development of LSIL to invasive carcinoma with increasing age. Ranabhat et al.[11] found Eighty percent of all the epithelial abnormalities were found in the age group > 40 years of age. The average age of patients for all the epithelial abnormalities was 49 years. Mishra et al. [12] also has pointed that in out of 36484 women studied, 12681 women were above 40 yrs of age in which 10.1% had SIL and 1.3% had Ca cervix.

The inflammatory smear was found to be more common in premenopausal age group women than postmenopausal age group women in this study. The STDs namely Trichomonas vaginalis, Candida albicans and bacterial vaginosis were also diagnosed in the cytology smears, and was more among the pre menopausal women. Out of 578 premenopausal women studied, 24 women reported inflammatory smear with infections (4.2%) like bacterial vaginosis (0.6%), Trichomoniasis (0.5%) and Candidiasis (2.6%). In post menopausal age group 164 women (41%) had inflammatory smear without any underlying pathology, STDs like candidiasis was seen in 9 (2.2%) cases and bacterial vaginosis was seen in 7 (1.8%) cases. This is in concordance with Ranabhat et al. [11] where women of age group between 21 to 80 were studied and in which 26% reported inflammatory smear, 1% reported candidiasis, 0.45% had Trichomoniasis, 7.6% had Bacterial vaginosis and 0.34% had Herpes simplex virus infection.

Ranabhat et al. [11] also showed Atypical Squamous Cells of Undetermined Significance (ASCUS) 2 %, Atypical Glandular Cells (AGC) 2%, LSIL 3 %, HSIL 6% and Squamous carcinoma 2% in which 75 %(1.5%) of ASCUS was in postmenopausal age group, no AGC was reported in postmenopausal age group, 83.3% (2.5%)of LSIL was in postmenopaual age group, 66.4%(4%) of HSIL and the 100% (2%) of squamous cell carcinoma were in the postmenopausal age group which also favours the present study.

In Misra et al. [12] cervical biopsy was taken in all cases of serious dysplasia and the cytology report indicated 100% similarity with histopathology. Cervical biopsy was performed in only 182 of the 219 cases of straight to the point carcinoma cervix diagnosed on cytology. The compatibility among histology and cytology was 100%. However, 29 fresh cases of cervical carcinoma were reported on histology. The cytology reports in these 29 cases had been inflammatory in 21 and inadequate smear in 8. The percentage of false negatives with cytology, for diagnosis of invasive carcinoma of cervix, was thus 10.3%.

Though conventional PAP smear was used in this study, various studies have demonstrated significant relationship between conventional PAP smear, Liquid based cytology (LBC) and Colposcopy method. Karimi-zarchi et al. has compared the results of conventional PAP smear, Liquid based cytology and Colposcopy method and found that Colposcopy with sensitivity of 70.9% can be stated to be superior than LBC PAP with sensitivity of 55.3% and in the comparison of compliance of the conventional PAP smear with that of the LBC, no significant relationship was found [13]. Thus, general colposcopy method has a higher sensitivity in diagnosis of any cervical lesions compared to the conventional PAP smear and liquid-based PAP smear.

## Conclusions:

Squamous intraepithelial lesions are more common in the postmenopausal age group compared to the premenopausal age group. Screening by PAP smear is essential in both the groups for early detection and management of epithelial abnormalities. We recommend at least a single life-time PAP screening cytology of the uterine cervix of all the women aged 40 to 50 years as there is a rapid increase cervical cancer mainly among in postmenopausal age group women. We propose that larger studies are required to estimate the pattern of cervical cytological abnormalities along with detection of common HPV strains in cervical cancer in Indian population. This knowledge would be useful for prevention of HPV infection with adequate intervention.

## Figures and Tables

**Table 1:** Distribution of lesions of cervix in pre menopausal and postmenopausal women N=978

PAP smear findings	Pre menopausal age group	Post menopausal age group	Total
Normal	252 (25.7%)	139(14.2%)	391 (40.0%)
Inflammatory Smear Without Underlying Pathology	292 (29.9%)	164(16.8%)	456(46.7%)
Inflammatory smear with infections :	24 (2.5%)	16 (1.6%)	40 (4.1%)
Bacterial vaginosis	06 (0.6%)	07 (0.7%)	13 (1.3%)
Trichomoniasis	03 (0.3%)	00 ( 0% )	03 (0.3%)
Candidiasis	15 (1.6%)	09 (0.9%)	24 (2.5%)
*LSIL	6(0.6%)	20 (2.1%)	26(2.7%)
**HSIL	02 (0.2%)	06(0.6%)	08 (0.8%)
***AGC	0	01 (0.1%)	01 (0.1%)
Squamous Cell Carcinoma	2 (0.2%)	13 (1.3%)	15(1.6%)
ATROPHIC SMEAR	0	41(4.2%)	41(4.2%)
	578(59%)	400 (41%)	978(100%)
*LSIL- Low Grade Squamous Intra epithelial Lesion; **HSIL- High Grade Squamous Intra epithelial Lesion; ***AGC - Atypical Glandular Cells
